# Geospatial mapping and data linkage uncovers variability in outcomes of foot disease according to multiple deprivation: a population cohort study of people with diabetes

**DOI:** 10.1007/s00125-019-05056-9

**Published:** 2019-12-17

**Authors:** Joanne E. Hurst, Ruth Barn, Lesley Gibson, Hamish Innes, Sicco A. Bus, Brian Kennon, David Wylie, James Woodburn

**Affiliations:** 1 School of Health and Life Sciences, Glasgow Caledonian University, Cowcaddens Road, Glasgow, Scotland G4 0BA UK; 2 Institute for Infrastructure & Environment, University of Edinburgh, Edinburgh, Scotland UK; 3 Amsterdam University Medical Center, University of Amsterdam, Department of Rehabilitation, Amsterdam Movement Sciences, Amsterdam, the Netherlands; 4 Queen Elizabeth University Hospital, Glasgow, Scotland UK; 5 Renfrewshire Health and Social Care Partnership, Paisley, Scotland UK

**Keywords:** Amputation, Diabetes, Foot, Geospatial mapping, Mortality, Social deprivation, Ulcer

## Abstract

**Aims/hypothesis:**

Our aim was to investigate the geospatial distribution of diabetic foot ulceration (DFU), lower extremity amputation (LEA) and mortality rates in people with diabetes in small geographical areas with varying levels of multiple deprivation.

**Methods:**

We undertook a population cohort study to extract the health records of 112,231 people with diabetes from the Scottish Care Information – Diabetes Collaboration (SCI-Diabetes) database. We linked this to health records to identify death, LEA and DFU events. These events were geospatially mapped using multiple deprivation maps for the geographical area of National Health Service (NHS) Greater Glasgow and Clyde. Tests of spatial autocorrelation and association were conducted to evaluate geographical variation and patterning, and the association between prevalence-adjusted outcome rates and multiple deprivation by quintile.

**Results:**

Within our health board region, people with diabetes had crude prevalence-adjusted rates for DFU of 4.6% and for LEA of 1.3%, and an incidence rate of mortality preceded by either a DFU or LEA of 10.5 per 10,000 per year. Spatial autocorrelation identified statistically significant hot spot (high prevalence) and cold spot (low prevalence) clusters for all outcomes. Small-area maps effectively displayed near neighbour clustering across the health board geography. Disproportionately high numbers of hot spots within the most deprived quintile for DFU (*p* < 0.001), LEA (*p* < 0.001) and mortality (*p* < 0.001) rates were found. Conversely, a disproportionately higher number of cold spots was found within the least deprived quintile for LEA (*p* < 0.001).

**Conclusions/interpretation:**

In people with diabetes, DFU, LEA and mortality rates are associated with multiple deprivation and form geographical neighbourhood clusters.

**Electronic supplementary material:**

The online version of this article (10.1007/s00125-019-05056-9) contains peer-reviewed but unedited supplementary material, which is available to authorised users.

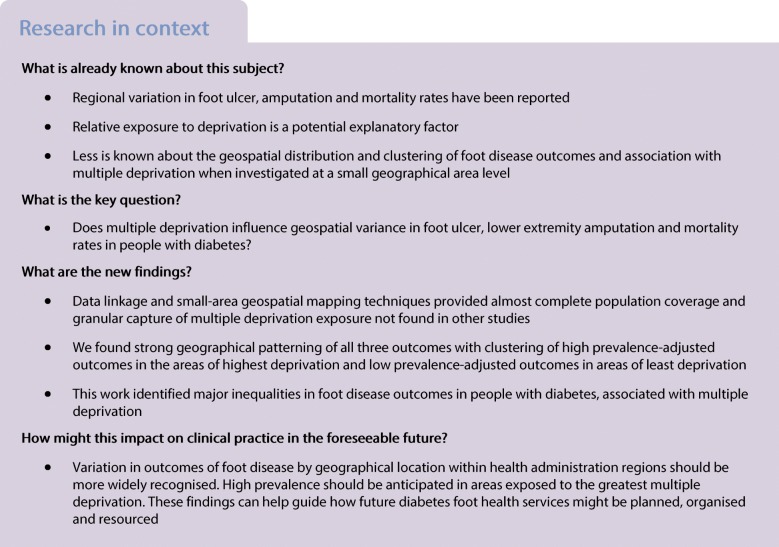



## Introduction

Diabetic foot ulcers (DFUs) are a devastating, disabling and costly complication of diabetes with a lifetime incidence between 19% and 34% [1]. Amputation-free survival rates are lower in individuals with healed or active ulcer history [2]. Further, mortality rates are high following lower extremity amputation (LEA), reportedly 44% at 1 year, 57% at 3 years and 77% at 5 years [3]. There are complex pathways leading to DFU and LEA and relative exposure to social deprivation is a potential risk factor [4–7]. With respect to LEA, exposure to deprivation together with high comorbidity burden, ethnicity, male sex and past history of DFU and LEA are strongly associated with increased risk [4]. Moreover, social deprivation increases the mortality burden in people with diabetes who develop a DFU: 14% per quintile of deprivation as reported by Anderson et al (2017) [8].

Exposure to deprivation is associated with increased risk of DFU, LEA and mortality through delays in seeking health assessment, financial pressures to maintain income and avoid cost of care, inappropriate advice and limited access to primary care advice and expertise, self-care, nutrition and footwear [6, 7]. However, this remains controversial when studied by geographical location. In UK cities with high levels of deprivation, socioeconomic status emerged as an independent predictor for developing a DFU [6]. Conversely, other UK studies have found no increase in RR of DFU for socioeconomic status and, once a DFU occurs, the effect of social deprivation exposure appears to be less influential with respect to independently predicting ulcer wound healing [9–12].

Relative exposure to social deprivation may be one factor that explains geographical variations in foot disease outcomes in people with diabetes [7]. For example, geographical variation has been demonstrated for rates of LEA in people with diabetes across National Health Service (NHS) England at region, health authority and primary care trust levels [13–15]. However, exposure to social deprivation could not explain these variations. This may be attributed to the large geographical areas studied comprising large populations and heterogeneity of levels of deprivation within each area. By contrast, in a US Medicare population, clustering of high incidence rates of LEA has been found in regions with lower socioeconomic status [16]. A more granular analysis identified a tenfold variation and geographical clustering in amputation rates between high income and low income and urban and rural regions of California [17]. Moreover, Bergin et al (2011) identified higher numbers of Australian state-level hospital-based episodes of care for diabetes-related foot disease in local government areas with the highest socioeconomic disadvantage [18]. However, geographical factors associated with accessibility to general practitioners or hospital clinics were not found to be associated with DFU or LEA in a large UK cohort study [6].

Thus far, the patterning of deprivation and its association with DFU, LEA and mortality rates have not been fully investigated. Reported studies measure exposure to deprivation by a single indicator (household income), or by multiple deprivation indices. Geographic unit of analysis varied from UK strategic health board and major regions to US hospital referral regions for all states or ZIP Code Tabulation Areas for the state of California. These geographic areas comprised inhabitant numbers of 3000 or higher. There were no studies that simultaneously investigated foot ulceration, LEA and mortality rates. Therefore, in this study we used a data linkage and geospatial mapping approach to investigate geographical variation of foot disease in Glasgow, UK, a city with high dispersion of social inequality [19].

## Methods

### Data sources

**Fig. 1:**
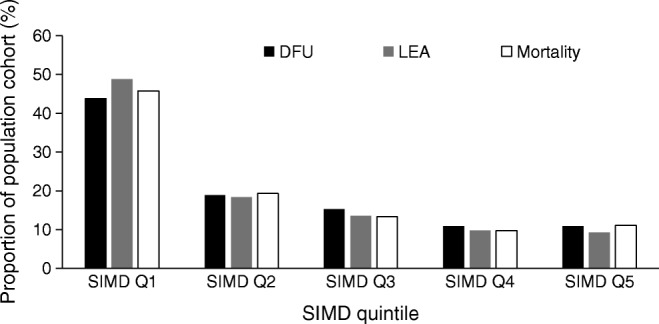
DFU, LEA and mortality preceded by DFU or LEA, by SIMD quintile (Q1–Q5)

We conducted a retrospective population cohort and geospatial mapping study within the NHS administrative area of Greater Glasgow and Clyde. Local and national health datasets were linked at the individual patient level using a unique patient identifier, the Community Health Index. Data linkage was undertaken by NHS Greater Glasgow and Clyde Safe Haven. Following linkage, the Community Health Index number was removed to anonymise information and databases were presented to researchers through a secure analysis platform (electronic supplementary material [ESM] Fig. 1).

The Scottish Care Information – Diabetes Collaboration (SCI-Diabetes) is a national population database containing demographic and clinical data from a fully integrated electronic patient record. This was linked to the national hospital admissions data (Scottish Morbidity Record SMR01) and the National Records of Scotland (NRS) data. We obtained peer review, Safe Haven review, Local Privacy Advisory Committee and Caldicott Guardian approvals for the study (reference: GSH/16/DI/002).

### Study population

Data extraction was undertaken in November of 2016. We included all people in the SCI-Diabetes database registered within NHS Greater Glasgow and Clyde from 1 January 2002. This health administration area comprises six local authorities with a population of 1,169,110 (21.6% of the Scottish population).

### Data variables

From SCI-Diabetes, we described the population cohort by ascertaining age, sex, diabetes type, disease duration and ethnicity. Diagnosis of diabetes was based on practice systems (read-codes) and SCI-Diabetes diagnostic information webforms. DFU outcome was extracted from SCI-Diabetes data items based on the foot risk stratification described by Scottish Intercollegiate Guideline Network (SIGN) guideline 116 criteria for previous or active ulcer on the right and left foot [20]. From SMR01, we extracted LEA outcomes using the Office of Population Censuses and Surveys (OPCS) classification of interventions and procedures coding. We combined codes for major (above the ankle joint [OPCS codes X09, X10]) and minor (below the ankle joint [OPCS codes X10, X11]) amputations for right and left sides [21]. Mortality data were extracted from the NRS database. Exposure to social deprivation was by 2016 Scottish Index of Multiple Deprivation (SIMD) score [22]. SIMD identifies small-area concentrations of multiple deprivation using 38 indicators across seven domains (employment, income, crime, housing, health, education and access). Deprivation scores were ranked highest to lowest for 6976 areas or data zones across Scotland, each with a mean population of 760. NHS Greater Glasgow and Clyde comprised 1460 data zones and we expressed scores by quintiles, with quintile 1 representing the 20% most deprived and quintile 5 representing the 20% least deprived areas (ESM Fig. 2). Each person with diabetes was assigned a data zone during data linkage using the most recent census and postcode information from the NRS record.

### Statistical analysis

We describe the study population and its demographic and clinical characteristics using mean, SD, absolute values and percentages. Maps were generated to visually present the spatial distribution of foot disease outcomes across the health board data zones. This was undertaken using ArcGIS 10.4 Geostatistical Analyst (ESRI, Redlands, CA, USA). The geographic unit of analysis was the SIMD data zones determined from the Scottish Government 2016 SIMD map [22] (ESM Fig. 2). Participant data zone number was extracted and linked to health records matching the shape file for the 2016 SIMD map (ESM Fig. 1). Since the total population of people with diabetes for each data zone was known, the crude prevalence-adjusted rates for DFU and LEA and incidence rates for death preceded by a DFU or LEA were calculated. Complete geographical coverage of LEA and mortality rate data was reliable, since this was extracted from SMR01 and NRS sources and therefore analysed between 2002 and 2016. SCI-Diabetes was implemented in NHS Greater Glasgow and Clyde in 2002, with screening tools for the foot added later and progressively rolled out over the board. We therefore estimated that complete geographical coverage of DFU data could be reliably determined towards the end of 2011. Therefore, DFU prevalence-adjusted rates were estimated from a subset of the cohort for the period 1 January 2012 to 7 November 2016.

We explored spatial clustering at the small-area data zone level (comprising a mean of 760 people), and specifically that outcome events would be correlated at nearer locations with similar social deprivation than outcomes at locations further apart. A data zone with a high prevalence of DFU, LEA or mortality rates surrounded by data zones with high values may be a statistically significant hot spot. Each data zone adjusted prevalence is compared proportionately to the prevalence rates for the entire health board area. If the observed data zone prevalence is very different from the expected data zone prevalence, and is too large to be random, is it a statistically significant hot spot (or conversely a cold spot). We conducted spatial autocorrelation using Getis–Ord G_i_* [23]. The G_i_* statistic is a *z* score and assumed to be normally distributed. Clusters with a 90% or higher significance level from a two-tailed distribution indicate significant clustering of data zones. In designing the spatial model, first we conceptualised the spatial relationship variable, in that data zone polygons were of differing dimensions (larger towards the periphery, representing more rural data zones, and smaller heading centrally, more urban). Consequently, we used a theoretical fixed distance band technique to counterbalance this. Second, we corrected the model for polygons with shared boundaries. Here, contiguity edges and corners were selected in the spatial model ensuring that data zones with a shared edge or corner were treated as neighbours and entered separately for each calculation in the hot spot analysis. Third, the G_i_* *z* score was displayed through colour codes (red, hot spots; blue, cold spots) across the SIMD maps. The distribution of hot and cold spots across SIMD quintiles was analysed separately using a one-sample χ^2^ test. These analyses were conducted using IBM SPSS Statistics 23 (IBM Corp, Armonk, NY, USA). A *p* value <0.05 was considered significant.

## Results

We extracted and linked the records of 112, 231 people from SCI-Diabetes between 2002 and 2016. The mean age of the population cohort was 67.2 years (SD 15.4 years), 53% were male, 74.9% were of white Scottish/British ethnicity and 75.2% had type 2 diabetes (Table 1). Overall, 42.0% of individuals were in the most deprived quintile and 14.0% in the least deprived.

**Table 1:** Summary statistics for the population cohort (*n* = 112,231) from 2002 to 2016

Variable	Category/measure	*n* or mean	% or SD	Total *n*	Missing (%)
Age^a^	Years	67.2	15.4	112,231	0.0
Sex	Female	52,799	47.0	112,231	0.0
	Male	59,432	53.0		
Ethnicity	White Scottish/British	73,638	74.9	98,340	12.4
	All other	19,403	19.7		
	Not known	5299	5.4		
Diabetes type	Type 1	9130	8.3	110,126	1.9
	Type 2	82,794	75.2		
	Others (combined)	18,202	16.5		
SIMD quintile	1 – most deprived	46,549	42.0	110,820	1.3
	2	21,225	19.2		
	3	15,023	13.6		
	4	12,561	11.3		
	5 – least deprived	15,462	14.0		
History of LEA	No	110,724	98.7	112,231	0.0
	Yes	1507	1.3		
Mortality^b^	No	110,588	98.5	112,231	0.0
	Yes	1643	1.5		

^a^Age presented as mean (SD)

^b^Mortality recorded as death preceded by a history of DFU, LEA or both

From 2012 to 2016, the denominator changed to 85,667 individuals registered on SCI-Diabetes during this time period. One or more DFUs was identified in 3923 individuals, with a crude prevalence-adjusted rate of 4.6% (Table 2). Between 2002 and 2016, LEA was identified in 1507 (1.3%) individuals and 1643 (an incidence rate of 1.5 per 10,000 per year) individuals with a past history of DFU or LEA had died (Table 1). We identified a significantly higher proportion of individuals for each outcome in SIMD quintile 1 for DFU (χ^2^ [*df*] value, *p* value), χ^2^[4] 1429, *p* < 0.001), LEA (χ^2^[4] 825, *p* < 0.001) and mortality preceded by DFU or LEA (χ^2^[4] 786.0, *p* < 0.001) (Fig. 1).

**Table 2:** Summary statistics for the population cohort (*n* = 85,667) from 2012 to 2016

Variable	Category/measure	*n* or mean	% or SD	Total *n*	Missing (%)
Age^a^	Years	65.48	15.6	85,667	0.0
Sex	Female	39,726	46.6	85,185	0.6
	Male	45,459	53.4		
Ethnicity	White Scottish/British	65,430	79.7	82,050	4.2
	All other	11,777	14.4		
	Not known	4843	5.9		
Diabetes type	Type 1	7022	8.4	83,503	2.5
	Type 2	63,500	76.0		
	Others (combined)	12,981	15.5		
SIMD quintile	1 – most deprived	34,564	41.1	84,123	1.8
	2	15,918	18.9		
	3	11,410	13.6		
	4	9847	11.7		
	5 – least deprived	12,384	14.7		
History of DFU	No	81,744	95.4	85,667	0.0
	Yes	3923	4.6		

^a^Age presented as mean (SD)

**Fig. 2:**
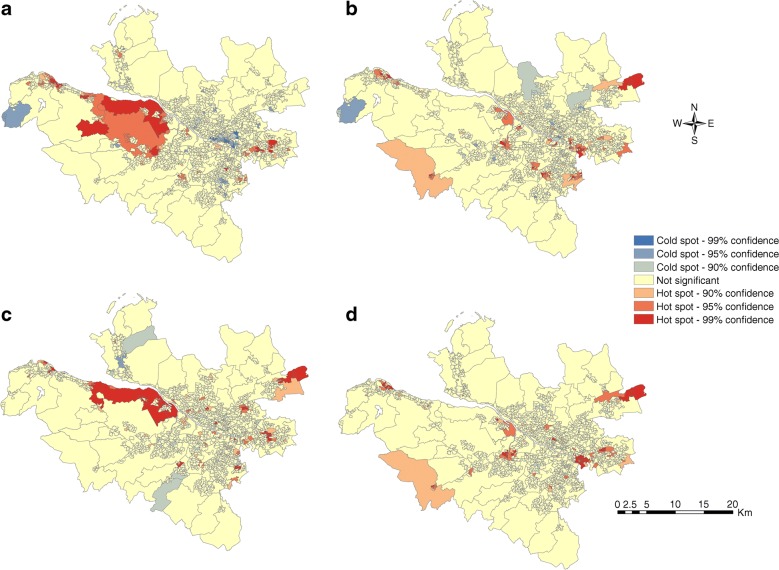
Geospatial maps identifying statistically significant hot and cold spots for (**a**) DFU, (**b**) LEA, (**c**) mortality preceded by DFU and (**d**) mortality preceded by LEA. In (**a**) and (**c**), the time period is from 2012 to 2016

**Fig. 3:**
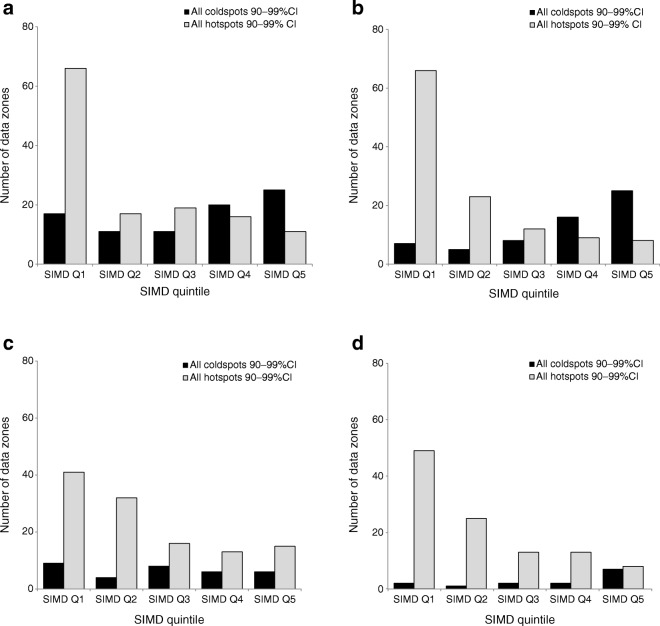
Proportion of hot and cold spot clusters by SIMD quintile (Q1–Q5) for (**a**) DFU, (**b**) LEA, (**c**) mortality preceded by DFU and (**d**) mortality preceded by LEA

Spatial autocorrelation identified statistically significant hot spot (high prevalence) and cold spot (low prevalence) clusters for DFU (2012–2016), LEA and mortality rates (2002–2016) (Fig. 2). In total, we identified 129 (8.8% of total 1460 data zones) hot spots and 84 (5.8%) cold spot data zones for DFU; 118 (8.1%) hot spots and 61 (4.2%) cold spot data zones for LEA; 117 (8.0%) hot spots and 33 (2.3%) cold spot data zones for rates of mortality preceded by a DFU; and 108 (7.4%) hot spots and 14 (1.0%) cold spot data zones for rates of mortality preceded by an LEA. Figure 2 provides a visual appreciation of clustering across the health board data zones. Neighbouring data zones show similarly high or low prevalence rates for all three outcomes and all diabetes. We found contiguous clustering of high prevalence data zones along the estuary of the River Clyde and in the east end of the city as well as the north-eastern local authority area. We found fewer cold spots but evidence of contiguous clustering in areas immediately bordering the north bank of the River Clyde and suburbs immediately to the north and south of Glasgow city centre. We identified a disproportionately high number of hot spots within SIMD quintile 1 (most deprived) for DFU (χ^2^[4] 79.6, *p* < 0.001), LEA (χ^2^[4] 101.2, *p* < 0.001), rates of mortality preceded by DFU (χ^2^[4] 26.4, *p* < 0.001) and rates of mortality preceded by an LEA (χ^2^[4] 50.7, *p* < 0.001). Although there were more cold spots forming in SIMD quintile 5 (least deprived) for each outcome, the trend was not statistically significant for DFU (χ^2^ 8.6, *p* = 0.071), rates of mortality preceded by DFU (χ^2^[4] 2.3, *p* = 0.680) and rates of mortality preceded by an LEA (χ^2^[4] 8.1, *p* = 0.086), but it was significant for LEA (χ^2^[4] 22.5, *p* < 0.001) (Fig. 3).

## Discussion

### Principal findings

We found a four-to-fivefold difference in the crude prevalence-adjusted rates of people with diabetes with DFU, LEA and mortality between the most and least deprived areas within NHS Greater Glasgow and Clyde. For DFU, over half of the hot spot clusters were found in the most deprived areas, with the remaining evenly distributed across quintiles 2–5. For LEA, three-quarters of the hot spots were distributed between the two most deprived quintiles, with 10% or fewer observed for SIMD quintiles 3–5. Similarly, death preceded by either a DFU or LEA was disproportionately observed in the two most deprived quintiles (66% and 62%, respectively). This health board comprised around one-fifth of the Scottish population. Our small-area geography maps effectively display and communicate these findings with sufficient granularity to detect near neighbour clustering. Relative exposure to deprivation does not prevent DFU, LEA or associated mortality rates, as we identified hot and cold spot clusters across all deprivation quintiles. Cold spots were equally distributed across all levels of deprivation for DFU and mortality rates but observed less often in the least deprived areas for LEA. The reasons for this are unclear, although around 80% of LEAs are preceded by a DFU and so less deprived areas might afford better secondary prevention and access to expert foot healthcare.

We have identified for the first time that neighbourhoods with poor outcomes related to foot disease are surrounded by a concentration of other areas with similar poor outcomes in the most socially deprived areas. This was noticeable along the Clyde estuary, in Inverclyde and in the east end of Glasgow city centre which, following deindustrialisation, has neighbourhoods within the top 5% of all deprived areas in Scotland. Such concentrations may serve to deepen poor health behaviours and culture and provide few resources to be drawn on, leading to the so-called ‘pull-down’ effect [24]. Conversely, but to a much lesser extent, we have identified lower than anticipated prevalent outcomes in local concentrations in the least deprived areas of the health board for LEA, the so-called ‘pull-up’ effect. We also found that local areas within SIMD quintile 1 (most deprived) had prevalent outcome rates similar to neighbourhoods with less deprivation. These deprived areas might draw on nearby diabetes foot services and other health resources. Further, people with diabetes might experience less social detachment and better behaviours such as self-esteem and resilience in these neighbourhoods, but this requires further investigation [19].

### Consistency with previous research

Regional variations in DFU and LEA rates have been previously reported but the association with social deprivation is controversial [13–16]. Our work confirms the association of ‘hot spot’ geographical patterning of LEA and socioeconomic deprivation and extends these findings to include DFU and mortality rates [17]. We employed SIMD, which captures a wider set of deprivation determinants including education, crime, housing and health, which builds in comorbidities, alcohol and drug misuse. Accordingly, SIMD is not directly comparable with other deprivation indices and when applied to small geographical areas might partly explain why other studies have not found a relationship. Nevertheless, it is possible to conceptualise how these domains can impact on foot disease in people with diabetes and their management. For example, non-attendance at general practice is highest in those in the most deprived areas of Scotland [25]. In people with diabetes, this could lead to unmet foot health needs including regular assessment of risk status and management strategies to prevent DFU. Using small-area maps to visualise DFU, LEA and mortality rates at a local level is novel, and follows work for type 2 diabetes risk in London [26].

### Strengths and limitations

The strength of the study lies in targeting Glasgow as a setting for the following three reasons:, (1) poor health outcomes are generally concentrated in West Central Scotland, in particular the Glasgow conurbation which is exposed to deindustrialisation and deprivation [27]; (2) major inequalities for diabetes incidence and mortality by socioeconomic status have been reported in Scotland, including Glasgow [28, 29]; and (3) DFU and LEA are highly prevalent in the Glasgow health board region [29]. Additionally, we used SIMD in local areas with similar deprivation characteristics. This beneficially limits heterogeneity in deprivation exposure and provides fine-grained geographical variability at local levels. There are a number of limitations. First, the use of crude rates of DFU, LEA and mortality is important for geospatial mapping to identify hot and cold spots. However, association with social deprivation may be confounded by other factors such as age and sex, and future explanatory analyses should make adjustments where indicated. Second, the study was conducted in one Scottish health board and may not be generalisable beyond that population. However, Glasgow’s unique SIMD patterning also provides an ideal test bed to assess the outcomes of foot disease in a region with high levels of deprivation, encompassing almost one-fifth of the Scottish population [19]. Ethnicity overlaps with poverty, deprivation and restricted access to healthcare [30]. Since key ethnic minority groups live in socioeconomically disadvantaged circumstances in the UK, this may have contributed to the geospatial patterns observed. We are unable to account for this with current geospatial mapping approaches. However, this is not straightforward because in Glasgow proportionately much higher key ethnic minority groups, including Chinese, Indian and Pakistani, live in much less socioeconomically disadvantaged circumstances [30]. Third, SCI-Diabetes captures routine clinical data that may vary in completeness and accuracy across locations and practices. Despite this, SCI-Diabetes is a validated clinical registry that captures 99% of individuals who have a diagnosis of diabetes within NHS Greater Glasgow and Clyde, enabling population-level analysis [31]. Fourth, for each person with diabetes, we extracted DFU, LEA and mortality data over a 14 year period from SCI-Diabetes and other datasets linked to their most recent data zone. Although data zones are constructed to represent natural communities and physical boundaries with stable and reasonably consistent population size, we are unable to account for potential misallocation of an outcome event to another previous data zone if a person moved. Neither are we able to account for DFUs prior to 2012, changes over time related to disease natural history or local variation in implementation and adoption of foot screening programmes and treatment pathways. Finally, although the results indicate an association between SIMD and the event outcome, due to the observational nature of this study, we cannot exclude reverse causality to explain these findings, i.e. that having a DFU or LEA results in increased exposure to deprivation rather than being a consequence of it.

### Summary

In conclusion, we successfully employed small-area geospatial mapping to reveal important inequalities in foot disease outcomes that are associated with multiple deprivation. There was four-to-fivefold variation in rates of DFU, LEA and mortality from the most- to least deprived neighbourhoods.

### Future research

We intend to extend the complexity of health board wide geospatial mapping and statistical modelling to include other explanatory factors such as ethnicity, comorbidity and access to foot healthcare services. These maps may help to widen engagement with local stakeholders, beneficiaries and policymakers. Moreover, these findings are important in guiding how future diabetes services might be planned, organised and resourced, taking socioeconomic disadvantage and geographical variation into account [8, 16]. Furthermore, changing care pathways locally using routine clinical data as provided by databases such as SCI-Diabetes may lead to reduced DFU and LEA incidence outside of study by conventional clinical trials [32].

## Electronic supplementary material

Figures (PDF 250 kb)

## Data Availability

The datasets accessed are not publicly available but access can be granted through NHS Safe Haven. The 2016 SIMD map shape file is available on the Scottish Government website: https://www2.gov.scot/Topics/Statistics/SIMD.

## Abbreviations

DFU: Diabetic foot ulcer
NHS: National Health Service
NRS: National Records of Scotland
LEA: Lower extremity amputation
OPCS: Office of Population Censuses and Surveys
SCI-Diabetes: Scottish Care Information – Diabetes Collaboration
SIMD: Scottish Index of Multiple Deprivation
