# Supplementary material for: Geospatial mapping and data linkage uncovers variability in outcomes of foot disease according to multiple deprivation: a population cohort study of people with diabetes
Source: Diabetologia. 2019 Dec 17;63(3):659–67. doi: 10.1007/s00125-019-05056-9 (PMC6997267; doi:10.1007/s00125-019-05056-9)
Supplement: Supplementary file 1 — (PDF 250 kb) [file 125_2019_5056_MOESM1_ESM.pdf]

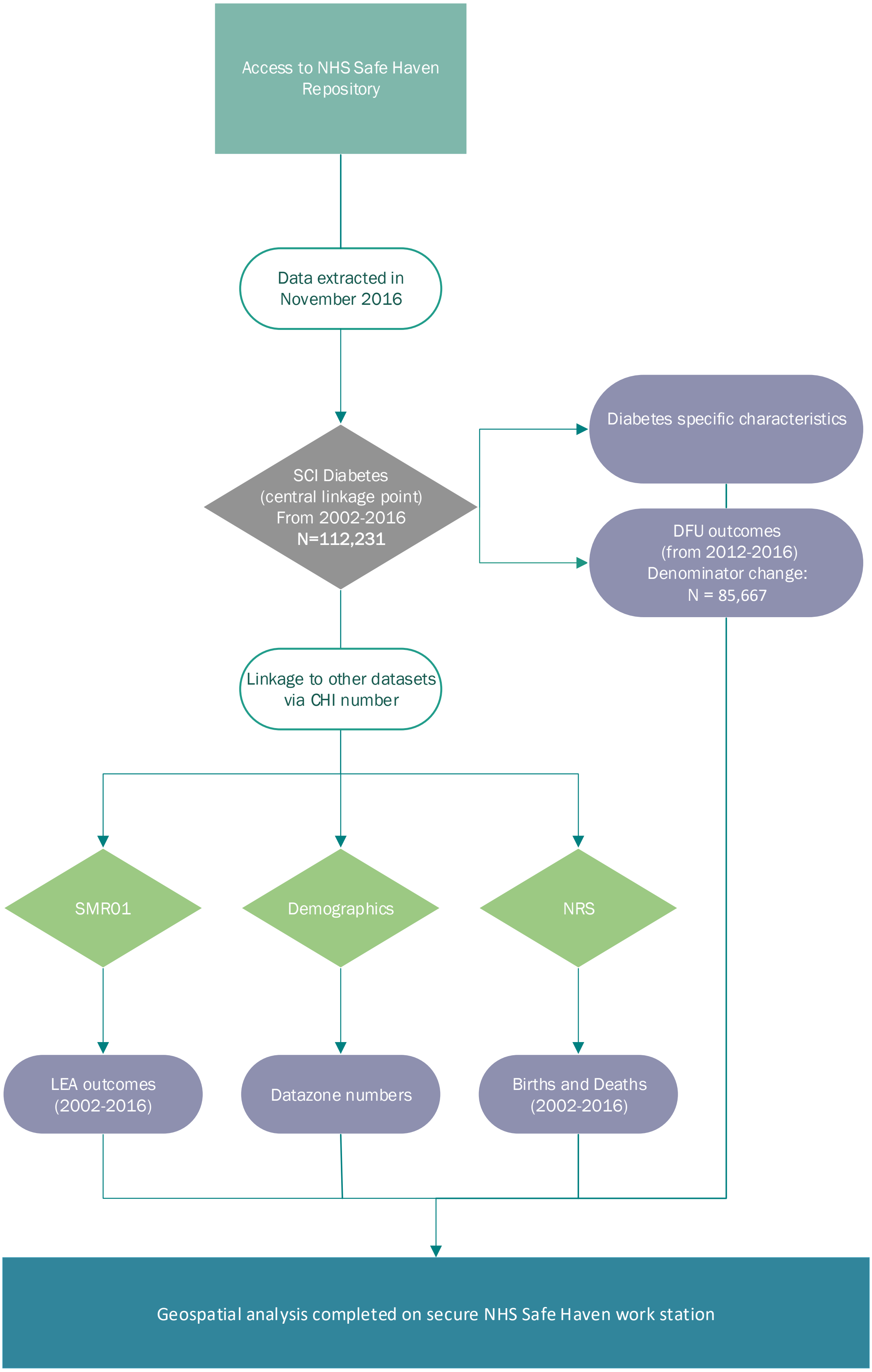


**ESM Fig. 1.** Extraction process from NHS Safe Haven indicating datasets accessed for geospatial analysis.

**(A)**

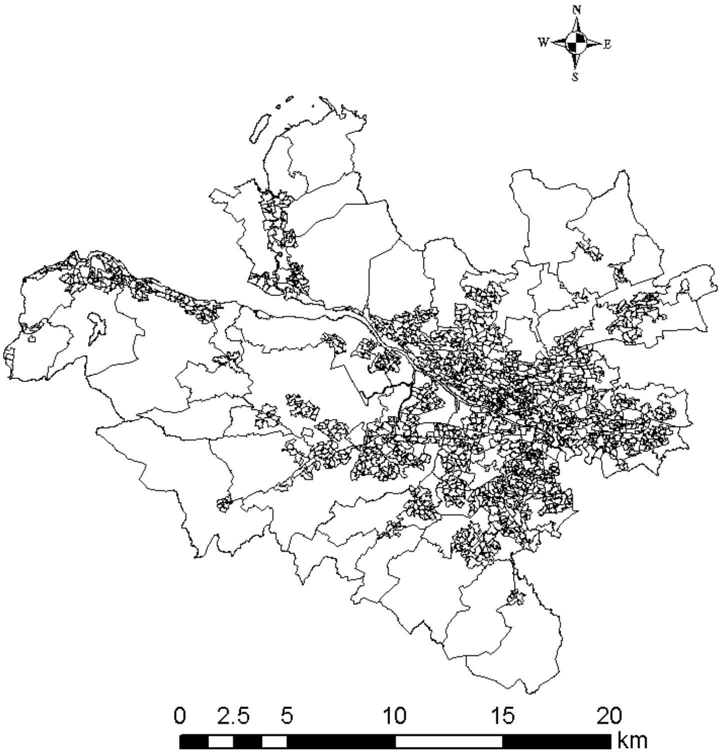


**(B)**

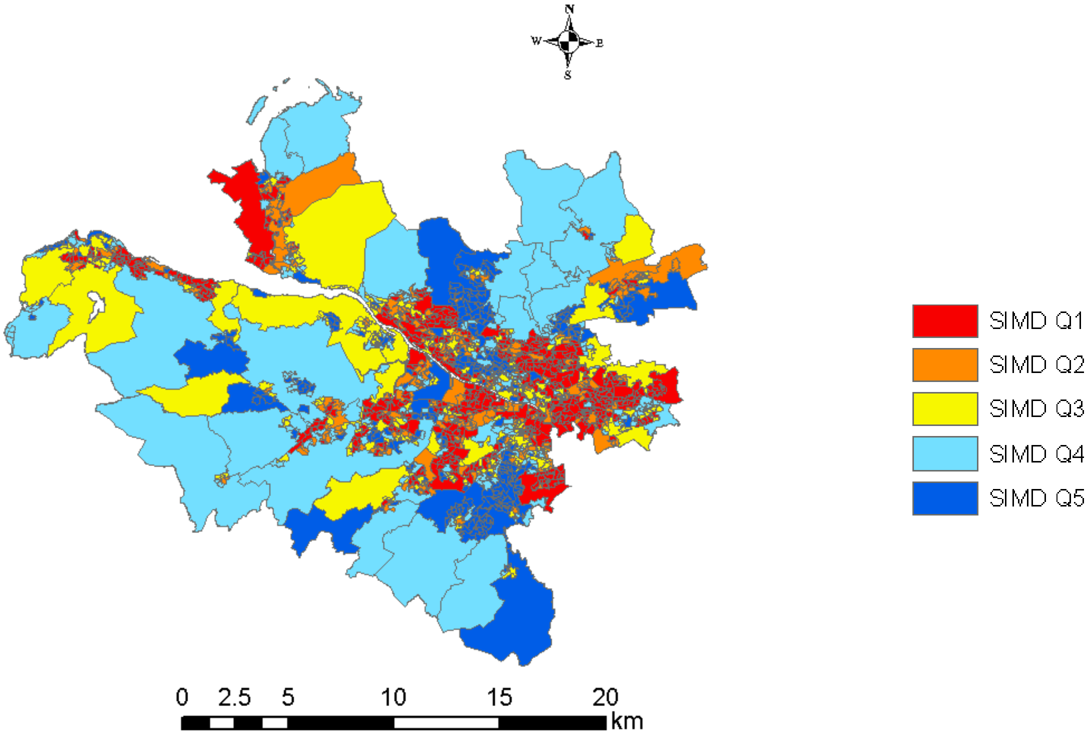


**ESM Fig 2. (A)** Base layer of map showing 1460 data zones within boundary of the health board **(B)** Choropleth map indicating SIMD quintile distribution across health board.
